# Serum zinc deficiency could be associated with dementia conversion in Parkinson’s disease

**DOI:** 10.3389/fnagi.2023.1132907

**Published:** 2023-04-27

**Authors:** Jieun Lee, Suyeon Park, Wooyoung Jang

**Affiliations:** ^1^Department of Family Medicine, Asan Medical Center, University of Ulsan College of Medicine, Seoul, Republic of Korea; ^2^Department of Biostatistics, Soonchunhyang University Hospital, Seoul, Republic of Korea; ^3^Department of Neurology, Gangneung Asan Hospital, University of Ulsan College of Medicine, Gangneung, Republic of Korea

**Keywords:** Parkinson’s disease, dementia, zinc, manganese, heavy metal, risk factors

## Abstract

**Background:**

Association between heavy metals and Parkinson’s disease (PD) is well noted, but studies regarding heavy metal levels and non-motor symptoms of PD, such as PD’s dementia (PD-D), are lacking.

**Methods:**

In this retrospective cohort study, we compared five serum heavy metal levels (Zn, Cu, Pb, Hg, and Mn) of newly diagnosed PD patients (*n* = 124). Among 124 patients, 40 patients were later converted to Parkinson’s disease dementia (PD-D), and 84 patients remained without dementia during the follow-up time. We collected clinical parameters of PD and conducted correlation analysis with heavy metal levels. PD-D conversion time was defined as the initiation time of cholinesterase inhibitors. Cox proportional hazard models were used to identify factors associated with dementia conversion in PD subjects.

**Results:**

Zn deficiency was significant in the PD-D group than in the PD without dementia group (87.53 ± 13.20 vs. 74.91 ± 14.43, *p* < 0.01). Lower serum Zn level was significantly correlated with K-MMSE and LEDD at 3 months (*r* = −0.28, *p* < 0.01; *r* = 0.38, *p* < 0.01). Zn deficiency also contributed to a shorter time to dementia conversion (HR 0.953, 95% CI 0.919 to 0.988, *p* < 0.01).

**Conclusion:**

This clinical study suggests that a low serum Zn level can be a risk factor for developing PD-D and could be used as a biological marker for PD-D conversion.

## Introduction

Parkinson’s disease (PD) is the second most common neurodegenerative disease after Alzheimer’s disease (AD) which typically affects sexagenarians or older people (Tysnes and Storstein, 2017). Starting from the development of levodopa in the late 1960s, several dopamine-based therapies have been invented, which have controlled distinctive motor features of PD, such as tremor, rigidity, and bradykinesia, to some extent (Abbott, 2010). On the other hand, non-motor symptoms, characterized by cognitive impairment, autonomic dysfunctions and psychiatric symptoms, have gained interest in the recent past for their hindering effect on patients’ quality of life during disease progression, but still are often unintentionally under-recognized in routine clinical practice (Chaudhuri et al., 2010).

PD’s dementia (PD-D), characterized by impaired attention, executive, and visuospatial dysfunctions, apathy, and hallucinations, is known to manifest at later stages (Hanagasi et al., 2017; Schapira et al., 2017). Cognitive impairment in PD increases the disease-related costs and caregiver burden, leading to higher institutionalization rates and mortality than in those PD patients without dementia (Hiseman and Fackrell, 2017; Mosley et al., 2017). The point prevalence of PD-D is assumed to be as much as 30%, and the cumulative incidence is reported to increase with disease duration (Aarsland and Kurz, 2010; Hanagasi et al., 2017). Several risk factors have been reported, such as old age, severe parkinsonism, and mild cognitive impairment, but no biological markers are yet to have significant predictive value (Levy et al., 2002; Janvin et al., 2005; Aarsland and Kurz, 2010).

Although 5%–10% of the variance in PD is thought to be explained by genetic etiology, the etiology of most sporadic cases is still unknown (>90%), suggesting a role for environmental factors in the occurrence of PD (Bjorklund et al., 2018; Deng et al., 2018; Ullah et al., 2021). Of that, heavy metals have gained attention among environmental factors due to increased exposure along with industrialization and their potential toxic influence on humans. Numerous epidemiological studies have reported relationships between long-term exposure to heavy metals and PD/PD-like symptoms. Most well known is probably Manganese (Mn), first described by John Couper in 1837, for its Mn-Induced Parkinsonism features in chemical factory employees (Guilarte, 2010). Normally, heavy metals are known for their free radical formation under the Fenton-Haber-Weiss reaction (Ball et al., 2019). These reactive oxygen species (ROS) induce oxidative stress, mitochondrial dysfunction, DNA damage, protein misfolding, and eventually apoptosis, resulting in neurodegeneration which is especially detrimental due to its limitation on recovery (Xie et al., 2012; Vellingiri et al., 2022).

However, not all heavy metals only have a detrimental influence on the body; metals such as manganese (Mn), iron (Fe), copper (Cu), and zinc (Zn) are classified as essential trace elements that act as cofactors for many enzymes, and the homeostasis of these elements is important because both deficiency and excess can result in various problems (Fraga, 2005). Furthermore, Uversky and Fink reported that some lower metal ion levels could accelerate the rate of α-synuclein (α-syn) fibril formation, a key pathologic substrate in PD pathogenesis, and Atarod et al. also revealed that different metal ions could trigger structural changes in α-syn fibrils and influence its cytotoxicity (Uversky et al., 2001; Atarod et al., 2022). Therefore, it could be hypothesized that heavy metal levels are crucial environmental factors for neurodegenerative diseases, including PD.

As outlined above, many studies have reported correlations between heavy metals and PD. However, little has been revealed about the link between heavy metals and non-motor symptoms, such as cognition. Considering that many studies indicate heavy metals could influence circadian rhythm, dementia risk, and psychological symptoms, we could postulate that serum heavy metals could also be associated with various non-motor symptoms in PD patients (Orisakwe, 2014; Parmalee and Aschner, 2017; Bakulski et al., 2020). In the present study, we examined five serum metals (zinc, copper, lead, mercury, and manganese) of 124 drug-naïve PD patients at the time of diagnosis, and then evaluated the correlation between baseline heavy metal levels and clinical characteristics of PD subjects. Next, we compared the serum heavy metal levels between PD-D and PD without dementia group. Finally, we examined the correlation between depletion or excess of these metals and PD-D development in drug-naïve patients.

## Materials and methods

### Patients and clinical assessment

We retrospectively enrolled 124 drug-naïve PD patients in the Movement Disorder Clinic of Gangneung Asan from January 2011 to November 2020 by reviewing the medical record. All PD patients were diagnosed according to the United Kingdom Parkinson’s Disease Society Brain Bank criteria, and the presence of relevant findings was revealed by [18F] N-(3-fluoropropyl)-2β-carbon ethoxy-3β-(4-iodophenyl) nortropane (FP-CIT) positron emission tomography. All subjects were followed longitudinally for at least 24 months after diagnosis and visited an outpatient clinic every 2 to 3 months for evaluation of motor and non-motor symptoms such as dementia on history and neurologic examination.

The exclusion criteria included the following: (1) prior or concomitant diagnosis of dementia at the time of PD confirmation; (2) co-morbidities that could affect cognitive function such as depression, cerebrovascular disease, normal pressure hydrocephalus, endocrine disease, alcohol overuse, uncontrolled DM, chronic kidney disease, and autoimmune disorders; (3) severe white matter change defined as grade 3 Modified Fazekas scale for white matter; (4) possibility of atypical parkinsonism, including progressive supranuclear palsy, multiple system atrophy, and corticobasal syndrome; and (5) suspicion of possible secondary parkinsonism, including vascular or metabolic parkinsonism, or PD due to toxic causes.

Clinical information, including age, sex, body weight, age of diagnosis, disease duration, and motor subtype which was classified into tremor dominant, intermediate, and akinetic rigid types, was obtained from medical records. We assessed initial motor severity at off status using the Unified Parkinson Disease Rating Scale (UPDRS)-III and Modified Hohen and Yahr (H&Y) stage and K-MMSE at the point of PD diagnosis. Levodopa equivalent dose (LEDD) 3 months after the initial diagnosis was calculated by using a previously published method (Tomlinson et al., 2010). Brain MRI was performed in all enrolled PD subjects at the time of PD diagnosis, and the Modified Fazekas scale for the white matter was evaluated. The study was approved by the ethical committee of Gangneung Asan Hospital.

### Assessment of the conversion of dementia

The time point of dementia conversion was defined as starting medication of cholinesterase inhibitors such as donepezil, rivastigmine, galantamine, and memantine. Before the initiation of medication, Korean version of the Frontal Assessment Battery (FAB-K) and K-MMSE were conducted in all PD subjects. Moreover, we also confirmed that all these subjects met the clinical criteria for diagnosing probable or possible PD dementia by a chart review from the Movement Disorder Society task force.

### Assay for heavy metal levels

Whole blood samples were collected in all enrolled PD subjects. This procedure was performed during hospitalization for workup of PD diagnosis, and samples were collected in the morning after overnight fasting. Collected samples were packed with ice and transferred to Eone Laboratories corporation (Incheon, South Korea), where the level of metals [zinc (Zn), copper (Cu), lead (Pb), mercury (Hg), and manganese (Mn)] was assayed by Inductively Coupled Plasma Mass Spectrometry (ICP-MS). Zn and Cu were measured by Agilent 7900 ICP-MS using serum acquired by centrifuging the whole blood sample at 200× g, and Hg, Mn, and Pb were measured by Agilent 7700 ICP-MS using whole blood. A 200-μL aliquot of sample was diluted (1,10) with 1,800 μL of 1% HNO3 and then centrifuged (701× g, 1 min). The assays were conducted following the standard guidelines provided by the manufacturer. To determine the level of contamination of elements from the collection tubes, a mock blood draw was performed using distilled water. Specifically, we collected and processed the distilled water using the same protocol and materials as the actual blood samples. We then analyzed the water samples for the presence of heavy metals using inductively coupled plasma mass spectrometry (ICP-MS). No significant contamination was observed in any of the distilled water samples, indicating that the collection tubes did not introduce any substantial levels of heavy metals into the blood samples. Trace Elements Serum and Metals Whole Blood from UTAK Laboratories Inc. were used as standard reference materials for quality control and assurance of Agilent 7900 and 7700. The inter-day and intra-day Coefficients of variation of all tests were <10%.

### Statistical analysis

Statistical analyses were performed using R version 4.1 and GraphPad Prism 9.0 (GraphPad Software, Inc., San Diego, CA). We adopted χ2 Tests for differences in categorical variables and independent *t*-tests for comparing continuous variables. We first compared the demographic, clinical parameters, and heavy metal levels of PD patients with and without dementia. Then, correlation analysis was used to affirm the relationship between heavy metal levels in significance and various clinical parameters of PD, such as the age of diagnosis, LEDD at 3 months, UPDRS-III, and K-MMSE. Additional correlation analysis was conducted to confirm heavy metal levels of PD-D and PD without dementia patients and clinical parameters. Cox proportional hazards regression analysis was also performed to identify factors associated with the time to dementia conversion in PD subjects. The variable selection used a stepwise method combining forward and backward selection. Finally, the Akaike information criterion was used to select the best model. The significance value was *p* < 0.05 for all the analyses.

## Results

### Demographic characteristics of PD-D and PD without dementia patients

The demographic characteristics of the participants are presented in Table 1. PD-D patients were older than non-dementia PD patients, including their age at diagnosis (79.50 ± 7.92 vs. 71.49 ± 9.50 years, *p* < 0.01; 72.05 ± 8.15 vs. 65.88 ± 9.67 years, *p* < 0.01). PD-D patients also had more severe clinical parameters than non-dementia PD patients, including longer disease duration, higher UPDRS-III scores, LEDD at 3 months, and modified H&Y scale (93.93 ± 24.16 vs. 72.08 ± 26.34 months; 29.08 ± 8.30 vs. 20.69 ± 8.78; 729.55 ± 122.38 vs. 564.77 ± 195.15 mg; 2.24 ± 0.34 vs. 1.77 ± 0.57, all *p* < 0.01). PD-D patients showed lower baseline K-MMSE scores and higher Fazekas scale (21.35 ± 3.64 vs. 26.11 ± 4.05, *p* < 0.01; 1.33 ± 0.69 vs. 0.83 ± 0.51, *p* < 0.01). Finally, PD-D patients were more prevalent in the akinetic rigidity subtypes group (*p* < 0.01).

**Table 1:** Comparison of basic demographic, clinical parameters, and serum heavy metal level between PD dementia and PD without dementia.

	Parkinson’s disease without dementia conversion (*n* = 84)	Parkinson’s disease with dementia conversion (*n* = 40)	*p*-value
Age	71.49 ± 9.50	79.50 ± 7.92	<0.01
Gender (male/female)	40/44	15/25	0.39^*^
Weight (kg)	62.02 ± 11.00	60.71 ± 10.45	0.53
Disease duration (month)	72.08 ± 26.34	93.93 ± 24.16	<0.01
Observation duration (month)	65.68 ± 23.85	58.85 ± 25.34	0.16
Type of PD (TD/intermediate/AR)	48/17/19	11/11/18	<0.01^*^
Age at diagnosis	65.88 ± 9.67	72.05 ± 8.15	<0.01
UPDRS-III	20.69 ± 8.78	29.08 ± 8.30	<0.01
LEDD at 3 months	564.77 ± 195.15	729.55 ± 122.38	<0.01
Modified H&Y	1.77 ± 0.57	2.24 ± 0.34	<0.01
K-MMSE	26.11 ± 4.05	21.35 ± 3.64	<0.01
Fazekas scale	0.83 ± 0.51	1.33 ± 0.69	<0.01
**Heavy metals**			
Zinc (μg/dL)	87.53 ± 13.20	74.91 ± 14.43	<0.01
Copper (μg/dL)	99.05 ± 20.82	99.16 ± 20.23	0.98
Lead (μg/dL)	1.82 ± 0.98	1.79 ± 0.91	0.86
Mercury (μg/L)	2.87 ± 2.04	2.71 ± 2.30	0.71
Manganese (μg/L)	11.08 ± 3.54	10.93 ± 4.03	0.84

These values represent the mean with the standard deviation or the number of subjects. *Chi-squared test PD, Parkinson’s disease; UPDRS-III, United Parkinson’s Disease Rating Scale Part 3; LEDD, L-dopa equivalent daily dose; H&Y, Hoeh and Yahr stage; K-MMSE, Korean version of the mini mental state examination scale; TD, Tremor-dominant; AR, Akinetic-rigidity.

### Heavy metals and PD

**Figure 1:**
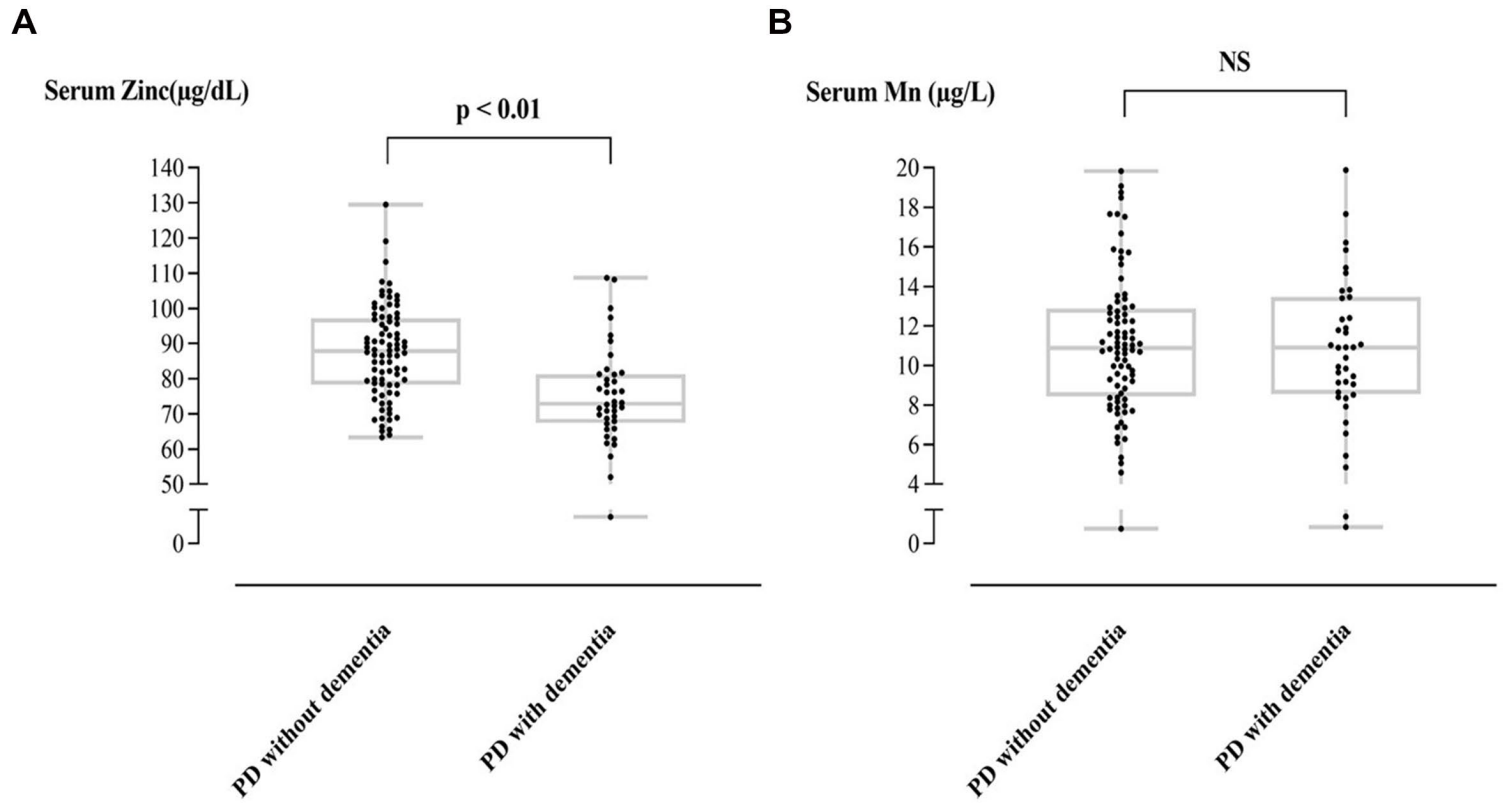
Individual T-tests of serum Zn **(A)**, Mn **(B)** level between PD with and without dementia.

Table 1 shows the heavy metal levels in PD-D and PD patients without dementia conversion. Of the five heavy metals, only Zn appeared to have a significant difference, with the former group showing a lower level of Zn than the latter (74.91 ± 14.43 vs. 87.53 ± 13.20 μg/L, *p* < 0.01; reference range: 81.0~121.0 μg/L). Mn level was above the normal limit (reference range: ≤8.0 μg/L) in both groups (11.08 ± 3.54 vs. 10.93 ± 4.03, *p* = 0.84), but it did not show a significant difference. Figure 1 compares the Zn and Mn levels between PD-D and PD patients without dementia.

**Figure 2:**
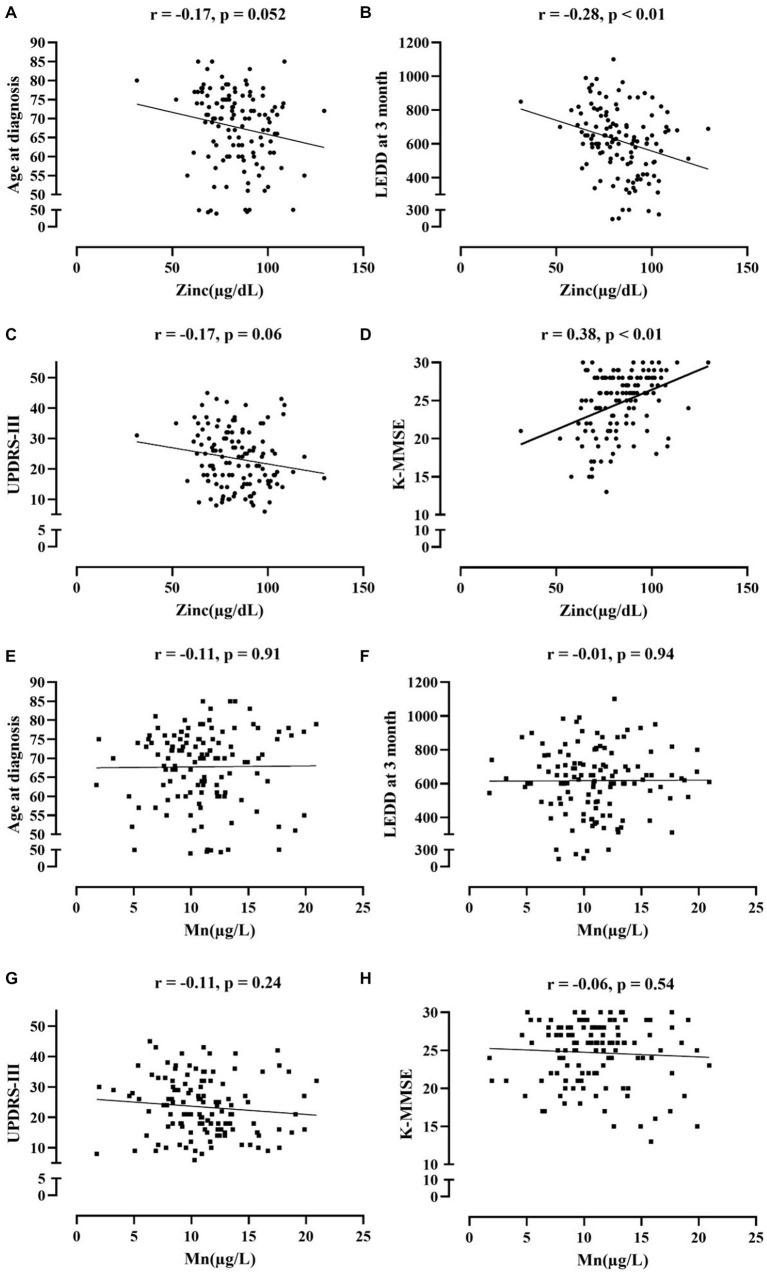
Correlation analysis between serum Zn **(A-D)**, serum Mn **(E-H)** and clinical parameters of PD.

In correlation analysis between Mn, Zn level and clinical parameters of PD adjusting age (Figure 2), Zn level was most strongly correlated with K-MMSE (r = 0.38, *p* < 0.01), followed by LEDD at 3 months (r = −0.28, *p* < 0.01), while there were no significant correlations between the Mn level and clinical parameters in PD subjects. UPDRS-III and age at diagnosis also showed negative correlation trends with Zn level, but it was not statistically significant (r = −0.17, *p* = 0.06; r = −0.17, *p* = 0.052). When classified into PD-D and PD without dementia groups, serum Zn was significantly correlated with K-MMSE among clinical parameters (r = 0.29, *p* < 0.01; r = 0.22, *p* < 0.05), while serum Mn did not have significant correlation with any parameters (Supplementary Table 1).

### Predictive factors for time to conversion of dementia in PD patients

Univariate and multivariate Cox regression hazard models were used to investigate risk factors, including Zn level, for the time to conversion of dementia in PD patients (Table 2). The univariate model revealed that akinetic-rigidity type of parkinsonism was most strongly associated with dementia conversion in PD subjects (HR 3.066, 95% CI 1.442–6.520, *p* < 0.01). Several clinical factors, including age, LEDD at 3 months, UPDRS-III, K-MMSE, and Fazekas scale, were also associated with time to dementia conversion as significant predictive factors of PD-D (HR 1.067, 95% CI 1.026–1.110, *p* < 0.01; HR 1.003, 95% CI 1.001–1.005, *p* < 0.01; HR 1.095, 95% CI 1.057–1.133, *p* < 0.01; HR 0.893, 95% CI 0.855–0.932, *p* < 0.01; and HR 2.515, 95% CI 1.538–4.111; *p* < 0.01, respectively).

**Table 2:** Univariate and multivariate cox proportional hazard regression analysis for predicting dementia conversion in PD subjects.

Covariates	Univariable analysis				Multivariable analysis			
	HR	95% CI		*p*-value	HR	95% CI		*p*-value
		Lower	Upper			Lower	Upper	
Age	1.067	1.026	1.110	<0.01				
Weight (kg)	0.990	0.957	1.024	0.55				
**Sex**								
Male	Ref							
Female	1.250	0.657	2.377	0.50				
**Type**								
TD	Ref							
Intermediate	1.862	0.805	4.303	0.15				
AR	3.066	1.442	6.520	<0.01				
LEDD	1.003	1.001	1.005	<0.01	1.002	1.000	1.004	<0.05
UPRDS-III	1.095	1.057	1.133	<0.01	1.071	1.033	1.109	<0.01
K-MMSE	0.893	0.855	0.932	<0.01	0.921	0.65	0.980	<0.01
Fazekas scale	2.515	1.538	4.111	<0.01				
Zinc (μg/dL)	0.972	0.936	1.009	0.14	0.953	0.919	0.988	<0.01
Copper (μg/dL)	0.997	0.983	1.010	0.62				
Lead (μg/dL)	0.940	0.646	1.369	0.75				
Mercury (μg/L)	0.919	0.777	1.089	0.33				
Manganese (μg/L)	0.955	0.850	1.073	0.44				

HR, Hazed Ratio; CI, Confidence Interval; UPDRS-III, United Parkinson’s Disease Rating Scale Part 3; LEDD, L-dopa equivalent daily dose; K-MMSE, Korean version of the mini mental state examination scale; TD, Tremor-dominant; AR, Akinetic-rigidity.

**Figure 3:**
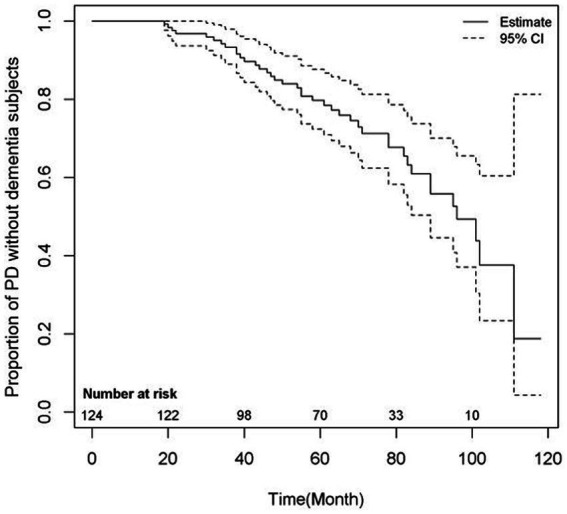
Risk of dementia conversion of PD patients.

In multivariate analysis, the final model selected LEDD at 3 months, UPDRS-III, K-MMSE, and serum Zn level for predicting the dementia conversion in PD patients (HR 1.002, 95% CI 1.000–1.004, *p* < 0.05; HR 1.071, 95% CI 1.033–1.109, *p* < 0.01; HR 0.921, 95% CI 0.650–0.980, *p* < 0.01; and HR 0.953, 95% CI 0.919–0.988; *p* < 0.01, respectively). Figure 3 shows the proportion of PD without dementia according to time.

## Discussion

This study reports two major findings. First, serum Zn level was significantly lower in the PD-D group compared with PD without dementia group at the time of diagnosis, and it also showed significant correlation with K-MMSE scores. Second, Zn deficiency contributed to a shorter time to dementia conversion in our study. To our knowledge, this is the first study confirming the longitudinal effect of Zn deficiency on dementia conversion in PD.

Although it remains unclear and inconsistencies persist, numerous studies indicate the role of heavy metals in the pathogenesis of PD (Bjorklund et al., 2018; Ball et al., 2019; Raj et al., 2021; Vellingiri et al., 2022). Several epidemiologic studies have revealed a correlation between exposure to these metals and the risk of PD. Dantzig reported that the blood mercury level was six times higher in PD patients, and chronic occupational exposure to Cu and Pb also were reported to increase PD risk in several studies (Kuhn et al., 1998; Gorell et al., 1999; Dantzig, 2006). However, there were also studies denying these correlations between heavy metals and PD risk, and these inconsistencies might be associated with different methodologies (Semchuk et al., 1993; Vieregge et al., 1995).

Chronic overexposure to Mn leads to Manganese-induced parkinsonism, known as “Manganism,” by accumulation of Mn in the basal ganglia circuit (Kwakye et al., 2015). Manganese-induced parkinsonism is similar to idiopathic PD in several clinical symptoms, including gait disturbance, masked face, and psychiatric changes. However, nonsignificant resting tremor and resistance to levodopa therapy combined with the absence of degeneration of dopaminergic neurons in the substantia nigra differentiate the two diseases (Guilarte, 2010; Kwakye et al., 2015). Still, Mn remains a potential risk factor for idiopathic PD, as several studies have shown elevated Mn levels in PD patients (Fukushima et al., 2010; Du et al., 2018). In our study, serum Mn level was elevated in both PD and PD without dementia groups, while serum Cu, Pb, Hg levels were within the normal reference range (70.0–155.0, ≤1.7, and <3.40 μg/dL, respectively). However, there was no correlation between serum Mn level and disease severity variables such as LEDD, UPDRS-III scores, and K-MMSE in our study. Therefore, the role of higher serum Mn levels in PD pathogenesis should be further clarified.

In our study, serum Zn level was significantly lower than the reference level in both PD-D and PD without dementia groups. Previous studies have reported that both excess and deficiency of Zn are thought to contribute to neurotoxicity, probably due to its complex mechanism in various enzymes and signaling pathways. Animal studies using PD models have shown that an overload of intracellular Zn cations and Zn treatment can degenerate nigrostriatal dopaminergic neurons, while *in vivo* experiments have shown that intracellular zinc chelators act as neuroprotectors to neurotoxins (Lee et al., 2009; Kumar et al., 2010; Tamano et al., 2019; Sikora and Ouagazzal, 2021). However, there are also studies showing conflicting results. While delicately regulated by Zn transporters and metallothioneins in normal conditions, recently, two meta-analyses regarding serum Zn level for its association with the risk of PD showed that reduced serum Zn level could be related to an increased risk of PD. In our study, Zn was also lower in PD patients, implying that Zn deficiency can be a risk factor for PD (Szewczyk, 2013; Du et al., 2017; Sun et al., 2017).

There are some studies which reported that serum Zn level was nonsignificant between PD and control patients (Kocatürk et al., 2000; Kim et al., 2018). For instance, Kim et al. recently reported a significant correlation between serum Cu levels and with risk of PD. In contrast, serum Zn level was not significantly different between the PD and control groups (Kim et al., 2018). However, the serum Zn level tended to be related to a lower dyskinesia risk in women, which indicates that the serum Zn level could be associated with the prognosis of PD. Regarding clinical prognosis, our results also showed a significant association between Zn level and many clinical parameters reflecting PD characteristics such as K-MMSE and LEDD at 3 months. There were also trends with UPDRS-III and age at diagnosis, but these associations did not reach statistical significance. Therefore, serum Zn level might also be serum biological markers reflecting clinical prognosis and PD occurrence. Notably, our study has collected blood samples taken from drug-naïve patients, to avoid confounding factors such as disease duration, medication effect, and heterogeneity of PD stage.

Many possible mechanisms could explain the association between Zn and PD. First, Zn is a key component of Cu, Zn-superoxide dismutase, which catalyzes superoxide to produce hydrogen peroxide and dioxygen, which are less harmful (Sea et al., 2015; Jarosz et al., 2017). Furthermore, Zn-metalloproteins are known to protect neurons from oxidative damage by releasing Zn ions in oxidative stress conditions and conducting their antioxidant actions (Ruttkay-Nedecky et al., 2013). Considering that oxidative damage to dopaminergic neurons in substantia nigra and consequent inflammatory reaction is considered an important pathologic mechanism of PD, increasing oxidative stress led by the reduction of Zn could contribute to PD pathogenesis (Ball et al., 2019). Second, mitochondrial dysfunction is the key factor of cell degeneration and is considered one of the central mechanisms in PD development (Bose and Beal, 2016). Disrupted mitochondria result in increased reactive oxygen species and could affect various cellular pathways leading to impaired intracellular components and cell death. Furthermore, many causative genes linked to familial PD are associated with various aspects of mitochondrial quality control, such as PINK1(PTEN-induced putative kinase 1) and Parkin (E3 ubiquitin ligase), which are known as familial PD-related gene products essential in the mitophagy process (Pickrell and Youle, 2015). Parkin has four ring domains, each coupled with two Zn2+ ions, and the removal of the Zn ion causes loss of function due to the unfolding of the protein (Seirafi et al., 2015). Saini and Schaffner suggested that Zn supplement could restore the condition of Parkin mutant Drosophila, which suggests that Zn deficiency could be associated with mitochondrial dysfunction (Saini and Schaffner, 2010). Therefore, mitochondrial dysfunction due to Zn deficiency may contribute to PD pathogenesis in increasing ROS and mitochondrial quality control. Third, the role of intracellular synaptic Zn level in PD pathogenesis has recently been emerging. *In vitro*, Zn was found to function as a potential neuromodulator, mainly for the N-methyl-D-aspartate (NMDA) receptor (Kay and Tóth, 2008; Amico-Ruvio et al., 2011). Pathological findings have revealed that synaptic Zn was densely deposited in the striatum and released along with glutamate at excitation. Zn modulates motor behaviors in harmony under normal conditions (Sikora and Ouagazzal, 2021). Considering that the degeneration of nigrostriatal neurons causes overactivation of glutamate projections to the striatum, excess intracellular Zn release combined with glutamate could accelerate the degenerative process. Sikora et al. reported the detrimental role of synaptic Zn ion promoting motor and cognitive deficits evoked by nigrostriatal dopaminergic denervation. On the contrary, a PD animal study showed that intracellular Zn ions might act as a protective factor by inhibiting the excessive NMDAR signaling that negatively impacts motor coordination and learning (Fantin et al., 2008; Sikora and Ouagazzal, 2021). Therefore, although more remains to be investigated, the synaptic, and intracellular function of Zn could be related to the development of PD.

Another important finding regarding serum Zn level is that the Zn deficiency group showed a significantly shorter time to dementia conversion.

Disruption of Zn homeostasis is well-known for its implication in various neurodegenerative disorders, including Alzheimer’s disease (AD) and PD. Ventriglia et al. conducted a meta-analysis investigating the alteration of serum Zn levels in AD and confirmed that the serum Zn level was significantly lower in AD patients compared with the control group (Ventriglia et al., 2015). Low serum Zn level in AD could be explained by Zn sequestration due to binding with amyloid-beta (Aβ; Sensi et al., 2018). Postmortem studies revealed that brain Zn accumulation could be a prominent characteristic in AD, combined with brain Aβ deposition (Religa et al., 2006). Although cortical and limbic Lewy bodies and Lewy neurites are considered pathologic features and cholinergic dysfunction is a more prominent feature in PD-D, there is an increasing number of studies revealing that there could be synergistic effects among α-syn, tau, and Aβ (Perez-Lloret and Barrantes, 2016; Jellinger and Korczyn, 2018). Irwin et al. suggested that AD-type pathology (Aβ and tau-containing neurofibrillary tangles) combined with α-syn could have a synergistic role in PD-D, and the level of AD pathology was found to have a positive correlation with cognitive impairment (Irwin et al., 2012, 2013). Therefore, considering the involvement of Zn in Aβ accumulation, zinc could also play a synergistic role in PD-D pathogenesis. Our finding that Zn deficiency could be associated with a shorter time of dementia conversion might support this hypothesis.

Zn in the brain is found mostly in the forebrain region, including the hippocampus, amygdala, olfactory bulbs, and frontal cortex, which are closely associated with memory and learning (Sikora and Ouagazzal, 2021). Current studies have revealed that along with cholinergic and cortical dopaminergic degeneration, increased α-syn accumulation in the basal forebrain and hippocampus, which overlaps the distribution of Zn, could contribute to PD dementia occurrence (Hall et al., 2014; Smith et al., 2019). Taken together, disruption of Zn homeostasis might contribute to the pathogenesis of PD-D, and the mechanism of Zn linked to PD-D must be further investigated.

This study has several limitations. First, it is a retrospective study, therefore the interval of the observation of the patients is heterogeneous. Patients might have been censored because of dementia development, and conversely, in those with regular clinical follow-ups, dementia might have been detected more easily. Both scenarios could have contributed to the over or underestimation of PD-D. Second, many previous studies that examined the relationship between heavy metal exposure and PD risk showed a correlation between chronic exposure and PD risk. However, our study estimated only the serum level at the diagnosis cross-sectionally. Therefore, there is a limitation in that the source or exposure duration was not reflected in this study. Third, for the definition of the dementia conversion time point, the initiation date of dementia medication with cognitive function tests at outpatient clinic visits was adopted. Therefore, it could be possible that the exact point of dementia occurrence was missed. In addition, we retrospectively confirmed PD-D conversion using diagnostic criteria for PD-D through chart review. However, a neuropsychological test was performed for each subject using different tools and at different time points, except for the K-MMSE and FAB-K. As a result, we were unable to investigate the characteristics of cognitive dysfunction and dementia using a consistent neuropsychological test. To supplement this diagnostic insufficiency, we added the subscores of the K-MMSE and FAB-K for patients who converted to dementia in Supplementary Table 2. Fourth, we could not exclude potential confounding factors, including an olfactory function test, non-motor symptoms such as RBD and freezing of gait, which are related to cholinergic dysfunctions, and regimen of dopaminergic treatments. These factors could have longitudinally affected dementia conversion, but it is not possible to estimate their impact. Lastly, this study is a single-center study in which the patient group is mostly located in rural areas and may have been affected by a specific environment. Further nationwide multicenter studies are required for the generalization of the results.

In conclusion, we demonstrated that PD-D patients had significantly lower Zn levels compared to PD without dementia groups. Furthermore, lower serum Zn level was significantly correlated with various clinical characteristics in PD patients, especially shorter time to dementia. These findings suggest that low serum Zn levels could be not only risk factors for developing PD but also one of the biological markers for predicting dementia conversion and disease subtyping. Therefore, a further prospective study is warranted to investigate the role of Zn in PD development and its pathogenesis.

## Data availability statement

The raw data supporting the conclusions of this article will be made available by the authors, without undue reservation.

## Ethics statement

The studies involving human participants were reviewed and approved by the ethical committee of Gangneung Asan Hospital. The patients/participants provided their written informed consent to participate in this study.

## Author contributions

WJ and JL proposed the research idea and performed the data analysis, interpretation, and wrote the manuscript. SP provided the clinical suggestions. WJ confirmed concepting and designing research and wrote the manuscript and prepared the manuscript for submission. All authors contributed to the article in meaningful manners.

## Funding

This research was financially sponsored by the Gangneung Asan Hospital Biomedical Research Center promotion fund. The funder was not involved in the study design, collection, analysis, interpretation of data, the writing of this article or the decision to submit it for publication. All authors declare no other competing interests.

## Conflict of interest

The authors declare that the research was conducted in the absence of any commercial or financial relationships that could be construed as a potential conflict of interest.

## Publisher’s note

All claims expressed in this article are solely those of the authors and do not necessarily represent those of their affiliated organizations, or those of the publisher, the editors and the reviewers. Any product that may be evaluated in this article, or claim that may be made by its manufacturer, is not guaranteed or endorsed by the publisher.
