# Supplementary material for: Serum zinc deficiency could be associated with dementia conversion in Parkinson’s disease
Source: Front Aging Neurosci. 2023 Apr 27;15:1132907. doi: 10.3389/fnagi.2023.1132907 (PMC10172503; doi:10.3389/fnagi.2023.1132907)
Supplement: Supplementary file 1 [file Data_Sheet_1.docx]

**Supplementary Table 1. Correlation analysis between clinical parameters and serum zinc and manganese levels in subgroups of PD patients, stratified by dementia conversion**

|  | Serum zinc | | | | Serum Manganese | | | |
| --- | --- | --- | --- | --- | --- | --- | --- | --- |
| Subgroup | PDD- | | PDD+ | | PDD- | | PDD+ | |
|  | r | p | r | p | r | p | r | p |
| Age at diagnosis | -0.12 | 0.27 | 0.04 | 0.83 | -0.04 | 0.75 | 0.14 | 0.40 |
| LEDD at 3 month | -0.14 | 0.19 | -0.16 | 0.33 | -0.05 | 0.66 | 0.21 | 0.20 |
| UPDRS-III | -0.03 | 0.78 | 0.06 | 0.70 | -0.09 | 0.43 | -0.15 | 0.36 |
| K-MMSE | 0.22 | <0.05^*^ | 0.29 | <0.01^**^ | -0.02 | 0.87 | -0.21 | 0.19 |

r: Pearson’s correlation coefficient. *p<0.05, **p<0.01

PD: Parkinson's disease; PDD-: Parkinson’s disease without dementia; PDD+: Parkinson’s disease dementia; LEDD: L-dopa equivalent daily dose; UPDRS-III: United Parkinson’s Disease Rating Scale Part 3; K-MMSE: Korean version of the mini-mental state examination scale

**Supplementary Table 2. FAB-K and K-MMSE scores of PD patients at the time of dementia conversion in the PD with dementia group.**

|  | PD with dementia (N = 40) |
| --- | --- |
| **FAB-K item** |  |
| Similarities | 1.95±0.69 |
| Lexical Fluency | 1.15±0.63 |
| Motor Series | 1.56±0.75 |
| Conflicting Instructions | 1.64±0.81 |
| Go-no Go | 1.54±0.60 |
| Prehension Behavior | 2.85±0.37 |
| Total Score | 10.85±2.61 |
| **K-MMSE item** |  |
| Orientation | 6.26±2.10 |
| Registration | 2.88±8.35 |
| Attention and Calculation | 1.77±1.22 |
| Delayed Recall | 0.90±0.75 |
| Language and Visuospatial construction | 6.59±1.07 |
| Total Score | 18.46±3.51 |

These values represent the means ± the standard deviation. FAB-K: Korean version of the Frontal Assessment Battery; K-MMSE: Korean version of the Mini Mental State Examination scale; PD: Parkinson's disease
